# A retrospective case study of successful translational research: Cardiovascular disease risk assessment, experiences in community engagement

**DOI:** 10.1017/cts.2024.529

**Published:** 2024-05-16

**Authors:** Michael E. Bales, Jifeng Zhu, Christine A. Ganzer, Farid Aboharb, Allegra Keeler, Krista A. Ryon, Brett J. Ehrmann, Julianne Imperato-McGinley, for the H2H Consortium

**Affiliations:** 1 Weill Cornell Clinical and Translational Science Center, New York, NY, USA; 2 Hunter-Bellevue School of Nursing, School of Health Professions, Hunter College, CUNY, New York, NY, USA; 3 Tri-Institutional MD-PhD Program, Weill Cornell Medicine, Rockefeller University, Memorial Sloan Kettering Center, New York, NY, USA; 4 School of Medicine, New York Medical College, Valhalla, NY, USA; 5 Department of Physiology and Biophysics, Weill Cornell Medicine, New York, NY, USA; 6 Division of Primary Care of the Weill Cornell Physician Organization, Weill Cornell Medicine, New York, NY, USA

**Keywords:** Community health partnerships, health disparities, cardiovascular diseases, diabetes

## Abstract

In underserved communities across New York City, uninsured adults encounter a greater risk of cardiovascular disease (CVD) and diabetes. The Heart-to-Heart Community Outreach Program (H2H) addresses these disparities by screening for CVD risk factors, identifying healthcare access barriers, and fostering community engagement in translational research at the Weill Cornell Medicine Clinical and Translational Science Award (CTSA) hub. Screening events are hosted in partnership with faith-based institutions. Participants provide a medical history, complete a survey, and receive counseling by clinicians with referrals for follow-up care. This study aims to quantify H2H screening participant health status; identify socioeconomic, health access, and health-related barriers disproportionately promoting the onset of CVD and diabetes; and develop long-term community partnerships to enable underserved communities to influence activities across the translational research spectrum at our CTSA hub. The population served is disproportionately non-white, and uninsured, with many low-income and underserved individuals. The program was developed in partnership with our Community Advisory Board to empower this cohort to make beneficial lifestyle changes. Leveraging partnerships with faith-based institutions and community centers in at-risk New York City neighborhoods, H2H addresses the increasing burden of diabetes and CVD risk factors in vulnerable individuals while promoting community involvement in CTSA activities, serving as a model for similar initiatives.

## Introduction

With 600,000 uninsured residents [1], New York City (NYC) has the largest uninsured urban population in the United States. Uninsured residents encounter a greater risk of developing cardiovascular disease (CVD) and diabetes [2]. Over the past decade, CVD has been the leading cause of death in NYC, and diabetes has often ranked in the top five causes of death [3]. This is partly the result of multiple barriers facing medically underserved communities (populations that do not have adequate access to healthcare [4]). These barriers, which include social, cultural, and economic health determinants, have disproportionately affected vulnerable communities. In recent years, the coronavirus disease 2019 (COVID-19) pandemic has further exacerbated systemic inequities in healthcare access [5].

In 2018, healthcare spending and lost productivity due to CVD exceeded $400 billion [6], making it the costliest disease in the United States. Despite decades of steady decreases in overall CVD risk, racial, geographic, and socioeconomic health disparities persist among specific subgroups in NYC. Screening for modifiable risk factors, such as high blood pressure (BP), obesity, diabetes, and elevated cholesterol, is critical for CVD risk reduction, especially through early intervention [7]. However, individuals in low-income and medically underserved communities often encounter barriers in the healthcare system [8]. Given NYC’s large uninsured, underinsured, and underserved population, innovative community–academic partnerships help address these barriers and promote health equity [9]. Prevention and early intervention via lifestyle modification and/or first-line medications are successful and cost-effective – preventing an additional 3.2 cases of diabetes per 100 person-years in at-risk populations [10]. Consequently, relatively simple interventions, performed consistently and with appropriate follow-up, could prevent significant amounts of disease and disability in underserved populations.

The Weill Cornell Clinical and Translational Science Center (CTSC) [11,12], with two of its partners – NewYork-Presbyterian Hospital [13], and Hunter-Bellevue School of Nursing [14] – initiated the Heart-to-Heart Community Outreach Program (H2H) in 2010 to help address healthcare disparities contributing to CVD in underserved communities of NYC. Importantly, the H2H program was developed with input from the CTSC’s Community Advisory Board (CAB) and community partners. H2H brings the clinic to the community by providing free health screenings in NYC’s underserved and minority communities. Events are open to the public and hosted by the CTSC Community Outreach Core’s network of faith-based institutions and senior centers [15], considered equal partners in the program. This program has also facilitated the development of long-term partnerships that enable the continuation of community engagement initiatives at the CTSC.

The program’s overarching goal is to help participants understand their current health status, particularly by identifying those with diabetes, hypertension, obesity, and hyperlipidemia, and provide early intervention through on-site consultations and connections to resources for follow-up healthcare. The program also enables additional community engagement by establishing partnerships of trust that can be used to find collaborators for other health related projects.

In community-partnered research, it is important for all members of the team to be involved throughout the translational research process, including the creation, implementation, evaluation, and dissemination of the research [16,17]. Collaborative community partners are considered equals in the program and are included in strategic decision-making. The H2H executive board of students and faculty coordinates research activities and medical protocol decisions, while community leaders promote and host events, determining the dates and times of events based on community needs. Over the years, additional services such as nutrition counseling, ophthalmology screenings, and cancer screenings were added at the request of community partners. Furthermore, leaders from partner sites have become members of our CAB, co-investigators on research projects, and partners for other CTSC health projects, including COVID-19 vaccinations and combating the opioid epidemic.

The aims of this translational science case study are to (1) quantify the health status of those participating in H2H screenings; (2) identify the socioeconomic, health access, and health-related barriers disproportionately affecting the program participants; and (3) develop long-term partnerships to enable community engagement and overcome barriers to health equity across the translational research spectrum at our Clinical and Translational Science Award (CTSA) hub.

## Screening protocol

This project was initially approved by the Weill Cornell Medicine (WCM) Institutional Review Board as a research study in 2010 and reclassified as a Quality Assurance/Quality Improvement project in 2023.

WCM team members obtain consent and pair participants with a student navigator who escorts them through the screening process. Participants provide self-reported demographics and medical history and complete a survey. A licensed clinician counsels participants on individualized CVD risk factors and provides educational materials on appropriate lifestyle and behavioral modification, along with resources for follow-up care. Uninsured participants or those without access to primary care services are given a list of free or sliding scale-based federally-qualified community health clinics or are referred to the Weill Cornell student-run free medical clinic. Individuals are not required to participate in the research survey to receive screening and counseling services.

Participants are assessed for height, weight, body mass index (BMI), BP, waist circumference, and biochemical measurements (blood glucose, hemoglobin A1c, and a complete lipid panel – high-density lipoprotein (HDL), low-density lipoprotein (LDL), total cholesterol, and triglycerides). A clinician counsels the participant, explains the results, and provides individualized follow-up information. Dieticians are frequently available to provide counseling when needed. Additionally, socioeconomic and demographic history questionnaires are completed to understand factors contributing to an increased disease burden in these populations.

Blood samples are taken by finger stick to perform a lipid profile (Cholestech LDX System) and assess hemoglobin A1c (HgbA1c; Bayer A1CNOW+ or Siemens DCA Vantage 2000) and blood glucose. BP, height, weight, and waist circumference are measured, and BMI is calculated. The student navigator records these results in a secure REDCap database [18] and provides the participant a paper copy to take home.

**Figure 1.**
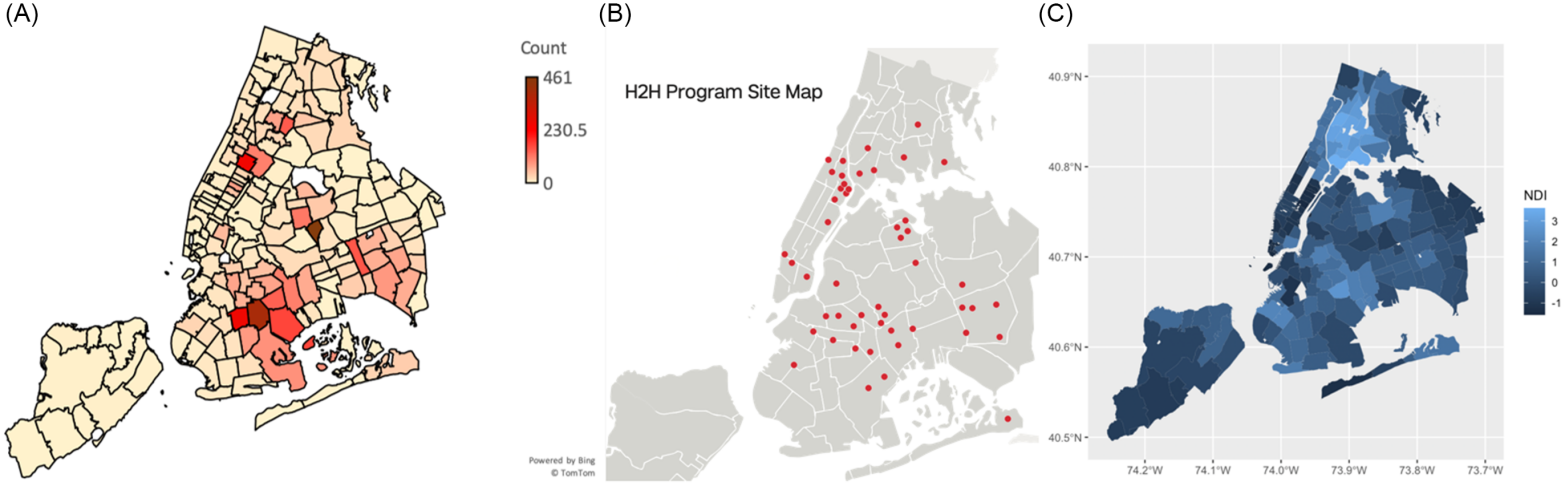
***a***. A thematic map showing the geographical distribution of H2H participants throughout the five boroughs of NYC. ***b***. A map of sites participating in the Heart-to-Heart Community Outreach Program (see acknowledgments for a list of sites). ***c***. A map of the Neighborhood Deprivation Index for NYC (light blue indicates underserved neighborhoods).

Demographic, anthropomorphic, and health screening data were aggregated and reported as summary statistics (counts, percentages, means, and standard deviations [SD]). Thematic maps were created by overlaying participants’ home ZIP codes as a density function atop an NYC public-domain map (see Fig. 1).

## Current status: implementation and dissemination

From 2010 to 2020, the program held 130 events and performed 5959 screenings at sites across NYC. Activities were temporarily suspended in 2020 due to the pandemic. Beginning in mid-2021, an abridged version of the screenings that minimized contact was offered at CTSC-supported COVID-19 vaccine sites. In mid-2022, the program began small-scale screenings with reduced capacity, and by 2023, all program activities were fully restored. Demographic, health status, and utilization of participants are summarized in Table 1.

**Table 1.** Demographics and healthcare utilization patterns of participants in the Heart-to-Heart Community Outreach Program

*Item*	*Count*	*Percent (%)*
***Number of events***	*130*	
***Number of screenings***	*5959*	
***Sex (n = 5834)***		
*Male*	*2148*	*36.8*
* Female*	*3682*	*63.1*
* Other*	*1*	*0.017*
* Prefer not to state*	*3*	*0.051*
***Age (n = 5774)***	***Mean***	***Standard dev***
	*54.3 years*	*39.6*
***Race (n = 5613)***		
* Black*	*3204*	*57.0*
* Latino*	*773*	*13.8*
* Asian*	*863*	*15.4*
* White*	*456*	*8.12*
* American Indian/Alaskan Native*	*44*	*0.8*
* Pacific Islander*	*19*	*0.3*
* Prefer not to state*	*72*	*1.3*
* Other*	*182*	*3.2*
***Ethnicity (n = 5593)***		
* Hispanic*	*995*	*17.8*
* Non-Hispanic*	*4598*	*82.2*
***Income (n = 4264)***		
* $0–$20,000*	*1378*	*32.3*
* $20,001–$50,000*	*1120*	*26.3*
* Over $50,001*	*1054*	*24.7*
* Decline to answer*	*712*	*16.7*
***Diabetes mellitus (n = 5842)***		
* At presentation*	*277*	*4.74*
* Previous diagnosis*	*917*	*15.7*
* If diagnosed, sub-optimal control? (Includes those on and off medication)*	*380*	*41.4*
* If diagnosed, on medication?*	*695*	*75.8*
***Dyslipidemia (n = 5699)***		
* At presentation*	*1651*	*29.0*
* Previous diagnosis*	*2149*	*37.7*
* If diagnosed, sub-optimal control? (Includes those on and off medication)*	*1335*	*62.1*
* If diagnosed, on medication?*	*1018*	*47.4*
***Hypertension (n = 5728)***		
* At presentation*	*1766*	*30.8*
* Previous diagnosis*	*2330*	*40.7*
* If diagnosed, sub-optimal control? (Includes those on and off medication)*	*1819*	*78.1*
* If diagnosed, on medication?*	*1737*	*74.5*
***Body mass index (n = 5829)***		
*Obese (BMI ≥30)*	*1985*	*34.1*
*Overweight (BMI 25–29.9)*	*2121*	*36.4*
*Normal weight (BMI 18.5–24.9)*	*1655*	*28.4*
*Underweight (BMI <18.5)*	*68*	*1.17*
***Emergency room usage in last 6 mo. (n = 1577)***		
*None*	*1342*	*85.1*
*1–2*	*208*	*13.2*
*3–5*	*21*	*1.33*
*>6*	*6*	*0.38*
***Healthcare utilization and potential barriers to access***		
*Have not seen a doctor in the last year (n = 5761)*	*1052*	*18.3*
*Avoided seeking medical care in the last year (n = 3260)*	*2003*	*61.4*
*Not US citizens or permanent residents (n = 5912)*	*2510*	*48.3*
***Insurance status (n = 5959)***		
*None*	*1698*	*28.5*
*Medicaid*	*194*	*3.26*
*Medicare*	*346*	*5.81*
*Private*	*501*	*8.41*
*Other*	*154*	*2.58*
*Yes, nonspecific*	*3066*	*51.5*

*Note*: H2H = Heart-to-Heart Community Outreach Program, LDL = low-density lipoprotein, HDL = high-density lipoprotein, BP = blood pressure.

While the total number of unique participants was 4300, the number of participants (n) for each variable changes due to repeat screenings and changes in questionnaire content at H2H screenings over time. For the first 6 years of the H2H program, participants were not asked to specify type of insurance. Incident cases (and suboptimal control of) diabetes: HbA1c > 7. Incident cases (and suboptimal control of) dyslipidemia: total cholesterol > 200, LDL > 130, HDL < 40, or triglycerides > 150. Incident cases (and suboptimal control of) hypertension: BP > 130/80.

### Aim 1: quantifying the health status of those participating in H2H screenings

With regard to the health status of the disadvantaged communities participating in H2H screenings, the mean age of participants screened was 54.3 years (SD 39.6). 63.1% were female and 91.9% of participants were non-white.

Disease prevalence revealed the following: 40.7% of participants were previously diagnosed with hypertension, with 78.1% having suboptimal control (BP > 130/80), even though 74.5% were on medication. 51.9% of participants screened had a HbA1c in the prediabetic or diabetic range [19] (HbA1c ≥ 5.7%). 15.7% of the participants were previously diagnosed with diabetes, with 41.4% having suboptimal control (defined as HbA1c > 7.0% [19]), although 75.8% of participants with known diabetes were on medication. 37.7% were previously diagnosed with dyslipidemia, and 62.1% had suboptimal control (total cholesterol > 200, LDL > 130, HDL < 40, or triglycerides > 150), even though 47.4% were on medication. 34.1% of participants were obese (BMI > 30).

**Figure 2.**
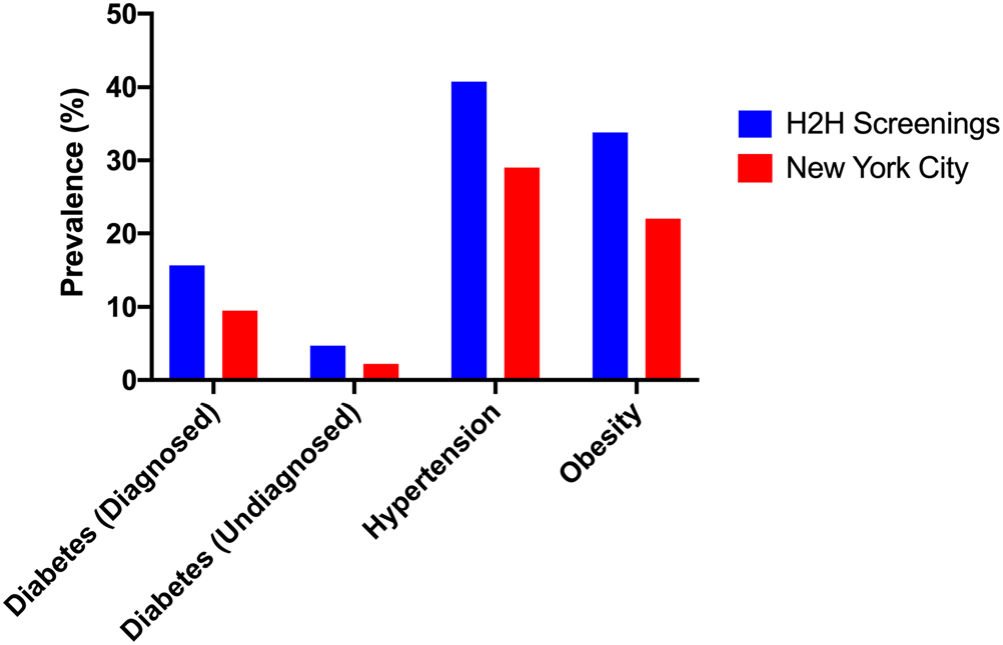
The bar chart compares the prevalence of these conditions based on statistics from the New York City Department of Health and Mental Hygiene for adults in New York City as a whole to that of Heart-to-Heart Community Outreach Program (H2H) participants. Undiagnosed diabetes refers to patients who were not aware or did not report having diabetes but who nonetheless met laboratory criteria for diagnosis.

Figure 2 compares the prevalence of selected conditions based on statistics from the NYC Department of Health and Mental Hygiene to that of H2H program participants.

For those without a previous diagnosis, 30.8% had hypertensive BP, 4.7% had elevated blood sugar (HbA1c > 6.5), and 29.0% had dyslipidemia at the time of screening.

### Aim 2: identifying the socioeconomic, health access, and health-related barriers disproportionately promoting the onset of CVD and diabetes

There were pronounced barriers to accessing medical care. (1) 18.3% of participants reported not seeing a doctor last year. (2) A majority (61.4%) reported avoiding medical care in the last year. (3) Annual income revealed that 32.3% reported <$20k, 26.3% between $20 and $50k, and 24.7% >$50k. 16.7% declined to answer.

28.5% of participants were uninsured, and 48.3% were not US citizens or permanent residents. In addition to this data, program leaders have observed that many individuals are not captured in the data due to an inability to provide consent, either because of language barriers or a refusal to answer survey questions. This group is heavily skewed toward immigrant or undocumented individuals with worries about revealing documentation status. A common remark from program participants is that the approximately 15 minutes they spend with H2H providers greatly exceeds the time they normally spend with their primary care provider. To explore these barriers further, the H2H program revamped the survey questions in 2023 to explore these topics.

### Aim 3: develop long-term partnerships to enable community engagement and overcome barriers to health equity across the translational research spectrum at our CTSA hub

Along with medical initiatives, the program has served as a cornerstone of our CTSA hub’s community engagement strategy. By having long-term outreach programs such as H2H, our CTSA hub can develop and maintain community partnerships that can last well beyond any single research project. Indeed, several of the original partners from 2010 and 2011 continue to host annual events to this day. This approach has resulted in the creation of a network of community partners where trust has already been established, providing several unique advantages. First, it allows the CTSA hub to quickly identify community partners to assist investigators with their research studies. Over the years, studies ranging from device trials to survey validation, from behavior interventions in minority youth to the National Institutes of Health (NIH) All of Us Research Program [20], have used H2H events to recruit underserved and minority individuals for their research studies. Due to the infrastructure for medical oversight and insurance coverage, investigators can initiate or even complete study activities on-site, thereby boosting participant completion rates for studies.

Second, this approach has enabled our CTSA hub to swiftly develop new projects based on community needs and rapidly evolving health crises. For example, in 2021, our CTSA hub launched a COVID-19 vaccination program in underserved communities across NYC that used H2H partners as vaccine sites and existing student volunteers as vaccinators [21]. This program was developed in response to a request by an H2H partner site and launched in under a month. From February 2021 to December 2021, the program administered nearly 27,000 vaccine doses across 40+ community sites to participants that were 69% African American or Hispanic [21].

Third, this approach has provided an entryway for community members to get involved in research activities at our CTSA hub. After successful H2H events, community leaders are often eager to engage in our Community Research Academy, a free course to train lay people on the research process, join our CAB, or get involved as a co-investigators on research studies.

### Limitations of the study

The major limitation of the program is that the demographic and health-related data collected are based on a convenience sample, a significant barrier to the application of rigorous experimental models. A second limitation is that the program has not yet incorporated protocols to track the progress of individual participants over time. The program is currently planning an expansion to implement this. Finally, a significant number of participants, primarily non-English speakers, could not provide informed consent to participate in the research study and did not have data collected.

## Further developments

To help mitigate language barriers, in 2023, the program simplified the consent process. In 2024, the program plans to translate the survey into commonly spoken languages at community partner sites and pair participants with bilingual volunteers.

Program leadership has also recognized that examining participant health outcomes via long-term follow-up will be a pivotal next step to refine and improve H2H screenings in the future.

### Implications for clinical practice, health education, public health policy, and future research

Based on their experiences interacting with the community over the past 12 years, program organizers have noted significant barriers to high-quality medical care. Many participants lack health insurance – a well-recognized problem in underserved communities. However, even among those insured, many perceived their routine interactions with primary care providers as suboptimal. The ability to speak extensively with an attending physician or nursing faculty member was a new experience for many participants.

From an institutional perspective, establishing a robust community-based participatory research network has required extensive effort and long-term commitment. However, by providing free health screenings, free health education, and other services to communities, trust and commitment have developed, enabling these communities to contribute to the translational research process at our CTSC hub. Specifically, community members have joined our CAB (which reviews and scores pilot grant applications) and become collaborators in research studies and projects with CTSC principal investigators. Thanks to the community network developed by the H2H program, many CTSC-supported investigators are now involved with underserved communities.

## Conclusion

The H2H program focuses on “bringing the clinic to the community.” By leveraging strong partnerships with faith-based institutions and community centers in at-risk NYC neighborhoods, the program breaks down barriers to engaging with the medical establishment. It helps to detect and address the increasing burden of diabetes and CVD risk factors in the most vulnerable individuals while simultaneously serving as a mechanism to develop long-term partnerships for community engagement activities at our CTSA hub.

Populations served by the H2H program have been disproportionately non-white, uninsured, low income, and underserved within the healthcare system. The burden of previously known CVD risk factors is high, and testing has revealed that many of these conditions have been newly discovered or poorly controlled. Participants faced numerous barriers to optimal healthcare utilization, including social, cultural, and economic health determinants. By fostering multidisciplinary and cross-institutional academic–community partnerships, the program has empowered individuals with a more detailed knowledge of their health status, facilitated positive lifestyle modifications, and provided access to medical advice, further addressing health risk factors, as well as promoting engagement of researchers with community members at all phases of the research process. By characterizing the community served by the initiative, we are now preparing a plan for the future that includes long-term tracking of participants and further exploration of barriers to healthcare access. Additionally, partnerships and trust developed through this program have enabled community involvement across our CTSA hub. We hope our experience will provide useful insights to those involved in similar initiatives.
